# Prevalence of intimate partner violence (IPV) during pregnancy in China: A systematic review and meta-analysis

**DOI:** 10.1371/journal.pone.0175108

**Published:** 2017-10-02

**Authors:** Tingting Wang, Yuan Liu, Zhanzhan Li, Kaihua Liu, Yang Xu, Wenpei Shi, Lizhang Chen

**Affiliations:** 1 Department of Epidemiology and Health Statistics, Xiangya School of Public Health, Central South University, Changsha, Hunan Province, China; 2 Department of Oncology, Xiangya Hospital, Central South University, Changsha, Hunan Province, China; 3 Department of Toxicology, Xiangya School of Public Health, Central South University, Changsha, Hunan Province, China; 4 Deyang Center for Disease Control and Prevention, Deyang, Sichuan Province, China; University of Ottawa, CANADA

## Abstract

**Background:**

Intimate partner violence (IPV) is the most common form of violence against women worldwide. IPV during pregnancy is an important risk factor for adverse health outcomes for women and their offspring. However, the prevalence of IPV during pregnancy is not well understood in China. The objective of this study was to estimate the pooled prevalence of IPV during pregnancy in China using a systematic review and meta-analysis.

**Methods:**

Systematic literature searches were conducted in PubMed, Web of Science, CNKI, Wanfang, Weipu and CBM databases to identify relevant articles published from the inception of each database to January 31, 2016 that reported data on the prevalence of IPV during pregnancy in China. The Risk of Bias Tool for prevalence studies was used to assess the risk of bias in individual studies. Owing to significant between-study heterogeneity, a random-effects model was used to calculate the pooled prevalence and corresponding 95% confidence interval, and then univariate meta-regression analyses were performed to investigate the sources of heterogeneity. Subgroup analysis was conducted to explore the risk factors associated with IPV during pregnancy.

**Results:**

Thirteen studies with a total of 30,665 individuals were included in this study. The overall pooled prevalence of IPV during pregnancy was 7.7% (95% CI: 5.6–10.1%) with significant heterogeneity (I^2^ = 97.8%, p < 0.001). The results of the univariate meta-regression analyses showed that only the variable “sample source” explained part of the heterogeneity in this study (p < 0.05). The characteristics “number of children” and “unplanned pregnancy” were determined as risk factors for experiencing violence during pregnancy.

**Conclusions:**

The prevalence of IPV during pregnancy in China is considerable and one of the highest reported in Asia, which suggests that issues of violence against women during pregnancy should be included in efforts to improve the health of pregnant women and their offspring. In addition, a nationwide epidemiological study is needed to confirm the prevalence estimates and identify more risk factors for IPV during pregnancy.

## Introduction

Intimate partner violence (IPV), including physical violence, psychological violence, forced sexual intercourse or other controlling behaviors, refers to violent behaviors committed by a partner in an intimate relationship and can cause physical, psychological or sexual injuries to the other person [1]. It is the most common form of violence against women worldwide [2–4]. According to the WHO, approximately 13~61% of females claim that they have experienced physical violence from their intimate partners before the age of 49 [2]. IPV can occur prior to pregnancy, during pregnancy and in the postpartum period. Because of the changes in the emotional, physical, social and economic needs of women during pregnancy, conception may be a time of unique vulnerability for women to become victims of IPV [5]. IPV during pregnancy is associated with non-fatal and fatal adverse health outcomes of pregnant women and their offspring. These adverse health outcomes may be caused by direct injuries of physical abuse to a gravida as well as physiological effects of stress from present or previous abuse on fetal growth and development [6]. Homicide[7] and suicide[8], which are fatal outcomes associated with IPV during pregnancy, are the two most extreme consequences. Non-fatal outcomes associated with IPV during pregnancy include adverse pregnancy complications (e.g., low birth weight [9, 10], premature delivery [11], miscarriage and abortion [12, 13], antepartum hemorrhage [14] and perinatal death [15]), negative health behaviors (e.g., drug and alcohol abuse, smoking and antepartum care procrastination [16]) and adverse psychosomatic outcomes (e.g., physical injuries [17], depression [18], anxiety [19] and suicidal tendencies [20]). With increasing knowledge of IPV during pregnancy, it has become an important public health issue [21].

The prevalence of IPV varies greatly among different regions. A WHO 19-country-study [22] based on household data from the Demographic and Health Surveys (DHS) and the International Violence Against Women Survey (IVAWS) showed that the prevalence of IPV during pregnancy was 3.8%~13.5% in Africa, 2.0%~5.0% in America, 1.8%~6.6% in Europe and 2.0% in Australia.

Antenatal care is the best chance to identify victims of IPV during pregnancy [22]. For many women in some remote areas, it may be the only time of contact with healthcare workers. In June 2011, the National Health and Family Planning Commission of the People’s Republic of China formulated and implemented the *Pregnant & Prenatal Care Regulation* and *Pregnant & Prenatal Care Standard* [23], which stipulated women to receive antenatal care at least five times during their pregnancy, and the number of visits should be increased as appropriate among high-risk pregnant women. However, it is difficult to achieve this goal in low-resource settings. Knowing the prevalence of IPV during pregnancy is the key step in helping to guide the formulation of health policy and allocation of resources, as well as the first step to developing and implementing effective interventions to prevent and treat associated sequelae. However, because of the scarcity of studies related to IPV during pregnancy across the country, we know little about the status of IPV during pregnancy in China. Therefore, the main purpose of this study was to estimate the pooled prevalence of IPV during pregnancy in China using a systematic review and meta-analysis.

## Methods

### Search strategy

We searched PubMed, Web of Science, China National Knowledge Infrastructure (CNKI), Wanfang, Weipu and China Biology Medicine disc (CBM disc) databases from the inception of each database to January 31, 2016 for articles reporting the prevalence of IPV during pregnancy in China. Search terms were as follows: domestic violence, family violence, partner violence, intimate partner violence, spousal violence, gender-based violence, pregnancy, prenatal, antenatal, prevalence, rate, magnitude, epidemiology, observational study and epidemiological investigation. The languages of these studies were restricted to Chinese and English. In addition, a manual search was performed of the reference lists of all articles selected in the first step. Two researchers (TTW and YL) independently completed the entire process.

### Selection criteria

In this study, an intimate partner referred to the past or present spouse, boyfriend, fiance, others living together or dating partners. The inclusion criteria were as follows: 1) an original epidemiological study in Chinese women; 2) samples obtained from clinical settings or general population or mixed; 3) studies with a clear survey time and place and within the pregnancy or one year after delivery at the time of assessment; 4) provided information about the sample size and prevalence estimation of IPV during pregnancy (or data to calculate these values); and 5) cross-sectional studies or the first evaluation of longitudinal studies. The exclusion criteria were as follows: 1) studies that did not report the prevalence of IPV during pregnancy or information adequate to evaluate it; 2) studies with partners as research subjects; 3) qualitative studies, case-control studies, case reports, reviews and conference presentations and abstracts; 4) studies with sample sizes < 100; 5) studies with incomplete or unclear data or logical errors; and 6) duplicate publications. In addition, if the same data were published in both English and Chinese, the paper published in Chinese was excluded.

### Data collection

Using a self-designed protocol, two reviewers (TTW and YX) extracted and evaluated the information from all included studies independently. The Cohen’ s kappa coefficient was calculated for measuring the inter-reviewer agreement according to the Cochrane Handbook [24], and disagreements were resolved by discussion or by consulting a third reviewer (LZC). The following information was extracted from each article: first author, year of publication, duration of data collection, geographic location, study design, sample source, sampling methods, sample size, investigating methods, measurement tools and number of victims of IPV during pregnancy (number of victims of physical violence, emotional violence and sexual violence were also collected if available). If those studies listed subgroup variables, such as family monthly income, marriage status, number of children, planned/unplanned pregnancy, baseline situation of the pregnant women and their partners (educational level, employment status, and alcohol consumption or tobacco consumption), the number of victims of IPV in those subgroups was collected independently.

### Risk of bias in individual studies

Two reviewers (ZZL and KHL) scored the quality of the included studies independently. The risk of bias in individual studies was assessed using the Risk of Bias Tool explicitly designed for the systematic review of prevalence studies developed by Hoy et al. [25]. The tool consisted of 10 items assessing the risk of bias in the following domains: selection bias (items 1–3), non-response bias (item 4), measurement bias (items 5–9) and bias related to the meta-analysis (item 10). For each criterion, the risk of bias was assessed as “low risk” or “high risk”. If the text was unclear, “high risk” was recorded. Discrepancies were resolved by discussion or by consulting a third reviewer (LZC). When more criteria were met in the included studies, the risk of bias was lower. A study was rated as having a low risk of bias if 8 or more items were met, a moderate risk of bias if 6 to 7 items were met, and a high risk of bias if 5 or fewer items were met.

### Statistical analysis

Before calculating the pooled prevalence, we performed normality tests for the original study rates and the transformed rates which were transformed using Log, Logit, arcsine and Freeman-Tukey double arcsine transformations [26]. Then, we determined whether the original rates should be transformed and which transformation method should be selected according to the test results. In the current meta-analysis, arcsine-transformed proportions were used. The pooled proportion was calculated as the back-transform of the weighted mean of the transformed proportions, using arcsine variance weights for the fixed-effects model and DerSimonian-Laird weights for the random-effects model. Owing to the expected significant heterogeneity across studies, a random-effects model was used to calculate the overall pooled prevalence and corresponding 95% confidence interval (CI). Heterogeneity between studies was evaluated using the Cochran’s chi-squared test and I^2^ statistic. The Cochran’s chi-squared test was used to assess whether the variation across studies was compatible with chance alone, and a p-value < 0.1 was used to represent statistically significant heterogeneity [24]. The I^2^ statistic, a quantitative indicator, was used to estimate the proportion of variance between studies due to statistical heterogeneity rather than chance (I^2^ ≤ 25% represents low heterogeneity, 26–50% represents moderate heterogeneity, 51–75% represents substantial heterogeneity, and 76–100% represents high heterogeneity) [24, 27]. To investigate possible sources of heterogeneity, univariate meta-regression analyses were performed according to the following variables: year of publication, sample size, geographic location, sample source, investigation methods and measurement tools. In particular, because of the large time span across the included studies, a random-effects meta-regression was used to explore trends over time with the year of publication as the covariate. Because there were a few significant variables, a multivariate meta-regression model was not constructed. Subgroup analyses were preformed based on the characteristics of the victims, perpetrators and pregnancy to explore the risk factors associated with IPV during pregnancy. Funnel plots and Egger’s linear regression tests were combined to assess potential publication bias, and a p-value < 0.1 indicated a significant difference. Sensitivity analyses were conducted in studies with a low or moderate risk of bias versus the overall included studies. All analyses were performed on R software version 3.2.3 (R Foundation for Statistical Computing, Vienna, Austria). PRISMA guidelines for systematic reviews and meta-analysis were strictly adhered to wherever appropriate (details see S1 Checklist) [28].

## Results

### Search results

**Fig 1:**
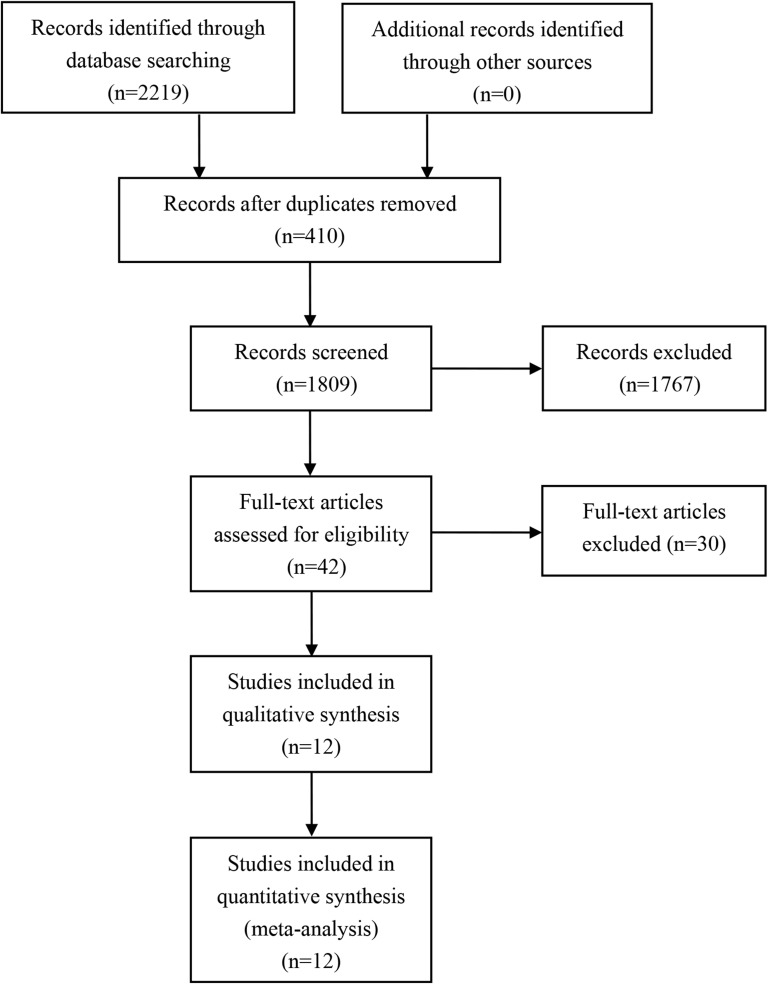
Flow diagram of included/excluded studies.

A total of 2219 studies were identified after an initial search (Fig 1). After removing duplicates and screening titles and abstracts, forty-two articles were potentially eligible and were reviewed in full text. After carefully reading these articles, Thirty studies were excluded (twelve duplicate publications, ten case-control studies, review papers and conference abstracts, three had data that were not extractable, one with partners as research subjects and one with the fifth year after delivery as the assessment time) (details see S1 File). Finally, a total of 12 studies [29–40] were included in the meta-analysis. The kappa score for screening titles and abstracts and assessing full-text articles was 0.78 and 0.81, respectively.

### Characteristics of identified studies

The characteristics of the included studies are shown in Table 1. The year of publication of the studies was between 1999 and 2016, within which nearly 83.3% (10/12 studies) [30–39] were from 2002–2008. The sample size of the included studies ranged from 200 to 12,044, with a total of 30,665 people. Most of the included studies (8/12 studies) were conducted in the mainland, whereas three studies [33, 39, 40] were in Hong Kong and one study [35] in Taiwan. Eleven studies were cross-sectional in design, and only one study [39] used a prospective cohort. Except for two studies [31, 37] based on the general population, the other studies were targeted at hospital populations. For the sampling method, more than 60% (8/12 studies) [30, 32, 33, 35, 36, 38–40] of studies used convenience sampling. Three studies [32, 39, 40] used the Chinese version of the Abuse Assessment Screen (AAS) questionnaire as the evaluation tool, one [33] used both the Chinese version of the AAS and the Chinese version of the Revised Conflict Tactics Scale (CTS2), one [35] used a physical abuse screening item derived from the AAS, and the others [29–31, 34, 36–38] used self-constructed items.

**Table 1:** Characteristics of studies on the prevalence of IPV during pregnancy in China.

No.	First author	Year of publication	Duration of data collection	Geographic location	Study design	Sample source	Sampleing method	Sample size	Inverstigation methods and measurement tools	IPV during pregnancy (n)	Prevalence (%)
1	Liu et al.	2016	2011.03–2012.02	Shenzhen	Cross-sectional	Hospital-based	Random sampling	7,820	Surveyed with self-constructed items filled out by the mothers	905	11.6
2	Zhang	2008	2006.10–2007.02	Hunan	Cross-sectional	Hospital-based	Convenience sampling	846	Surveyed with Chinese AAS filled out by the mothers	96	11.3
3	Huang	2008	2007.05 and 2007.12	Shanghai	Cross-sectional	Hospital-based	Convenience sampling	200	Surveyed with self-constructed items filled out by the mothers	5	2.5
4	Xu et al.	2008	2004.07–2007.12	Shenzhen	Cross-sectional	Population-based	Stratified cluster sampling	1,513	Face-to-face interview with self-constructed items	93	6.1
5	Lau et al.	2008	2002.07–2003.02	HongKong	Cross-sectional	Hospital-based	Convenience sampling	1,200	Surveyed with Chinese AAS and Chinese CTS2 filled out by the mothers	134	11.2
6	Yang et al.	2007	2003.01–2003.12	Taiwan	Cross-sectional	Hospital-based	Convenience sampling	1,143	Surveyed with selected AAS items filled out by the mothers	79	6.9
7	Fan et al.	2006	2004.03–2004.09	Henan, Guangdong	Cross-sectional	Hospital-based	Cluster sampling	2,835	Face-to-face interview with self-constructed items	327	11.5
8	Wu et al.	2005	2001.12–2002.02	Tianjing, Liaoning, Henan and Shanxi	Cross-sectional	Hospital-based	Convenience sampling	1,215	Face-to-face interview with self-constructed items	85	7.0
9	Guo et al.	2004	2001.11–2002.02	Tianjing, Liaoning, Henan and Shanxi	Cross-sectional	Population-based	Stratified cluster sampling	12,044	Face-to-face interview with self-constructed items	998	8.3
10	Guo et al.	2002	2002.03–2002.04	Beijing	Cross-sectional	Hospital-based	Convenience sampling	380	Surveyed with self-constructed items filled out by the mothers	29	7.6
11	Leung et al.	2002	2000.10–2001.02	HongKong	Prospective cohort study	Hospital-based	Convenience sampling	838	Face-to-face interview with Chinese AAS	87	10.4
12	Leung et al.	1999	1998.08–1998.11	HongKong	Cross-sectional	Hospital-based	Convenience sampling	631	Face-to-face interview with Chinese AAS	27	4.3

AAS = Abuse Assessment Screen Questionnaire; CTS2 = the Revised Conflict Tactics Scale.

### Risk of bias in individual studies

Of all included studies, 16.7% (2 studies) [33, 36] had a low risk of bias, 75% (8 studies) [29–32, 34, 35, 37, 39] had a moderate risk, and 16.7% (2 studies)[38, 40] had a high risk. None of the studies met all ten criteria. Half of the studies [30–32, 38–40] rated poorly for representativeness of the sample, whereas the same number of studies [29, 32, 35, 38–40] did not use an acceptable case definition. Fortunately, five studies [32, 33, 35, 39, 40] used the Chinese AAS or the Chinese AAS combined with the Chinese CTS2, which were validated in a Hong Kong Chinese population [41, 42], to measure the existence of IPV during pregnancy.

### Overall IPV during pregnancy

**Fig 2:**
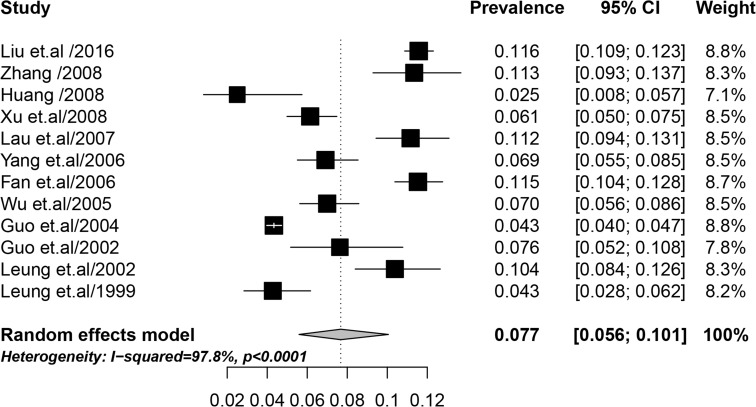
Forest plot of prevalence of IPV during pregnancy in China. The vertical dotted line indicates the overall effect size of all studies combined.

The forest plot in Fig 2 shows the data extracted from the single studies and on the overall pooled prevalence of IPV during pregnancy from the meta-analysis. The point prevalence of IPV during pregnancy reported in the original studies ranged between 2.5% and 11.6%. Using the random-effects model, an overall meta-analysis prevalence of 7.7% (95% CI: 5.6–10.1%) was obtained for IPV during pregnancy with substantial heterogeneity between studies (I^2^ = 97.8%, p < 0.001).

Notably, there was significant heterogeneity between studies included in the meta-analysis. To investigate and explain the possible sources of heterogeneity, univariate meta-regression analyses were performed (Table 2). Only the variable “sample source” explained part of the heterogeneity in this meta-analysis (p = 0.031), whereas the variables “geographic location”, “investigation methods” and “evaluation tools” all failed to explain the source of heterogeneity (all p > 0.05). Furthermore, there was no statistically significant line trend between the prevalence variation and year of publication (p = 0.208).

**Table 2:** Univariate meta-regression models.

Study characteristics		Estimate	SE	95% CI	*P*	Moderator analysis^a^		
						DLT (df)	*P*	VAF (%)
Year of publication		0.005	0.004	-0.003, 0.014	0.208	1.586 (1)	0.208	30.77
Sample size						0.340 (2)	0.844	21.15
	< 1,000 [reference]	-	-	-	-			
	1,000~2,000	0.034	0.059	-0.081, 0.150	0.560			
	>2,000	0.015	0.055	-0.093, 0.122	0.788			
Geographic location						0.033 (1)	0.856	7.69
	Hong Kong/Taiwan [reference]	-	-	-	-			
	Mainland	-0.009	0.047	-0.101, 0.084	0.856			
Sample source						4.628 (1)	0.031	65.39
	population-based [reference]	-	-	-	-			
	Hospital-based	0.068	0.031	0.006, 0.129	0.031			
Investigation methods						0.501 (1)	0.479	28.85
	Face-to-face interview [reference]	-	-	-	-			
	Fill out by mothers	0.026	0.037	-0.046, 0.098	0.479			
Measurement tools						0.866 (3)	0.834	15.39
	Chinese AAS [reference]	-	-	-	-			
	Chinese AAS and Chinese CTS-2	0.047	0.091	-0.132, 0.226	0.608			
	Selected item of the AAS	-0.028	0.091	-0.207, 0.152	0.726			
	Self-constructed items	-0.026	0.055	-0.133, 0.082	0.641			

DLT = DerSimonian-Laird test; VAF = variance accounted for.

^a^Compares model with no variables versus model with each variable separately via DerSimonian-Laird test to test the effect of each variable on the between-study heterogeneity and indicates the proportion of residual heterogeneity in the simplified model accounting for the heterogeneity in the full model.

Pooled prevalence estimates of all subgroups are presented in Table 3. Only the variables “number of children” and “unplanned pregnancy” had significant effects on the prevalence of IPV during pregnancy, demonstrating that women who had more than two children reported a significantly higher prevalence of IPV during pregnancy than women who had at most two children (10.8% vs 6.8%, p = 0.004), and the summarized prevalence in women with an unplanned pregnancy was significantly higher than in women with planned pregnancy (35.9% vs 11.6%, p = 0.032).

**Table 3:** Prevalence of IPV during pregnancy in different subgroups.

Category	Subgroup	No.of studies	event	n	Prevalence (%) (95% CI)	I^2^ (%)	P	χ^2^	P
Family monthly income								1.79	0.181
	Below/equal to local average	2	43	218	19.6 (14.6, 25.2)	19.5	0.265		
	More than local average	2	179	1,148	15.6 (13.6, 17.7)	0	0.798		
Marital status								0.98	0.323
	Married/cohabiting	4	796	14,294	9.9 (4.5, 17.2)	98.5	< 0.001		
	Single/divorced/separated	4	43	352	15.5 (7.4, 25.8)	79.5	< 0.001		
Number of children								8.24	0.004
	≤2	2	195	2,725	6.8 (4.8, 12.4)	77.3	0.035		
	>2	2	180	1,663	10.8 (9.4, 12.4)	0	0.751		
Unplanned pregnancy								4.59	0.032
	Unplanned	3	196	656	35.9 (13.6, 62.1)	97.7	< 0.001		
	Planned	3	128	1,035	11.6 (8.6, 15.1)	57.7	0.094		
Maternal education level								1.31	0.253
	≤9 years	4	670	6,157	15.9 (7.8, 26.2)	98.7	< 0.001		
	>9 years	4	922	14,682	10.0 (5.5, 15.6)	98.6	< 0.001		
Maternal employment status								0.49	0.485
	Unemployed	5	578	4,052	21.5 (13.0, 31.4)	95.9	< 0.001		
	Employed	5	686	6,044	16.7 (8.3, 27.3)	97.8	< 0.001		
Maternal drinking								1.19	0.276
	Drinker	5	194	1,917	20.7 (10.6, 33.2)	94.7	< 0.001		
	Non-drinker	5	731	12,959	12.9 (5.7, 22.4)	98.8	< 0.001		
Maternal smoking								1.63	0.202
	Smoker	5	112	859	22.0 (12.4, 33.4)	89.4	< 0.001		
	Non-smoker	5	813	14,019	13.1 (5.8, 22.8)	98.8	< 0.001		
Paternal education level								0.23	0.632
	≤9 years	3	191	2,630	18.1 (7.0, 32.9)	97.7	< 0.001		
	>9 years	3	496	10,780	11.9 (4.4, 22.3)	98.0	< 0.001		
Paternal employment status								0.98	0.323
	Unemployed	4	69	246	28.2 (7.6, 55.4)	92	< 0.001		
	Employed	4	325	2,564	15.6 (8.1, 25.1)	97	< 0.001		
Paternal drinking								2.16	0.141
	Drinker	4	821	11,703	15.2 (6.4, 26.8)	99.2	< 0.001		
	Non-drinker	4	771	9,429	7.0 (2.6, 13.1)	98.2	< 0.001		
Paternal smoking								1.08	0.299
	Smoker	3	534	9,309	15.8 (5.3, 28.8)	98.8	< 0.001		
	Non-smoker	3	153	4,100	7.9 (2.2, 16.7)	93.6	< 0.001		

### Simple subtypes of IPV

The pooled prevalence of simple physical violence, simple psychological violence and simple sexual violence were 3.6% (95% CI: 1.6–6.2%), 4.2% (95% CI: 1.8–7.5%) and 1.3% (95% CI: 0.6–2.5%), respectively, with significant differences among these types of violence (χ^2^ = 6.36, p = 0.042) (data not shown). Furthermore, multiple comparisons were performed. The results showed that there was a significant difference between the pooled prevalence of simple psychological violence and simple sexual violence (χ^2^ = 4.47, p = 0.035), whereas there were no significant differences between the pooled prevalence of simple physical violence and simple psychological violence (χ^2^ = 0.11, p = 0.744), as well as for simple physical violence and simple sexual violence (χ^2^ = 3.66, p = 0.056) (data not shown).

### Publication bias and sensitivity analysis

**Fig 3:**
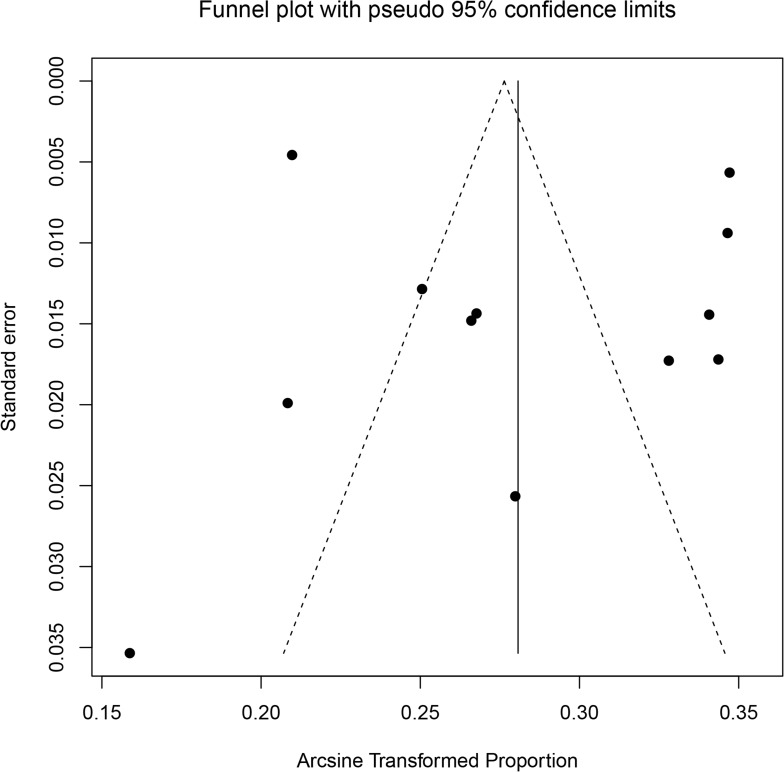
Funnel plot with 95% confidence limits of the prevalence of IPV during pregnancy in China. The solid line represents the summary effect estimates, and the dotted lines are pseudo 95% confidence limits.

Funnel plots and Egger’s test were combined to explore the potential publication bias in this meta-analysis. As shown in the Egger’s funnel plot of the twelve included studies (Fig 3), no evidence of obvious asymmetry was visually observed. Moreover, the t-score of for the plot was 0.521 (P = 0.614), which indicated that there was no evidence of significant publication bias in this meta-analysis.

Sensitivity analyses were conducted with the 10 studies that had a low or moderate risk of bias. The estimate of IPV during pregnancy in these studies (8.1%, 95% CI: 5.7–10.8%) was similar to the overall pooled estimate (7.7%, 95% CI: 5.6–10.1%), indicating the robustness of this analysis.

## Discussion

There are currently no nationwide data on IPV during pregnancy in China. In this study, we quantified the proportion of IPV during pregnancy in China using data from thirteen studies that met the inclusion criteria in our systematic review and meta-analysis and involved more than 30 thousand individuals. To our knowledge, this is the first time that the epidemiology of IPV during pregnancy in China was reviewed comprehensively using a meta-analysis.

The present meta-analysis showed that the prevalence of IPV during pregnancy is 7.7% (95% CI: 5.6–10.1%) in China, which is lower than the prevalence of IPV during last year among general Chinese females [43] as well as the specific prevalence estimates of IPV prior to conception [37, 40] and that in the postpartum period [31, 44, 45], suggesting that pregnancy may be a mitigating factor for IPV in China. However, there is a discrepancy between the available evidence regarding whether pregnancy causes a reduction in the prevalence of IPV [46]. A multi-national study on female victims of domestic violence conducted by the WHO [22] showed that most victims had previously experienced physical violence from their spouses prior to pregnancy. However, in Brazil, Serbia and Montenegro, approximately 50% of females experienced IPV for the first time during their pregnancy [2]. Further studies should be conducted to explore the role of pregnancy on the change in both the prevalence and patterns of IPV and the way in which pregnancy could influences women’s coping styles for violence. In addition, according to the WHO, the prevalence of IPV during pregnancy in the Philippines was 2.0%, Cambodia 2.8%, Azerbaijan 4.0%, and Jordan 5.0% [22], whereas another study reported that the prevalence in Japan and Thailand were 1% and 4%, respectively [2]. Therefore, we conclude that the prevalence of IPV during pregnancy in China is one of the highest reported in Asia.

Studies showed that the majority of women who suffered physical abuse during pregnancy also reported psychological abuse [16, 47, 48]. Moreover, in a review preformed in a worldwide study on the prevalence of violence against women during pregnancy from 1996 to 2010, psychological abuse was thought to be the dominant form of abuse during pregnancy in some cultures [49]. Both women who had and had not experienced violence during pregnancy reported higher rates of psychological aggression than that of physical assault or sexual coercion during pregnancy [48]. Compared to women without a history of psychological abuse, even in the absence of sexual or/and physical abuse, women with a history of psychological abuse during pregnancy were more likely to report a significantly poorer mental health-related quality of life [50]. In our study, the prevalence of simple psychological abuse (4.2%) was significantly higher than simple sexual abuse (1.3%) and higher than simple physical abuse (3.6%), although this was not significant, which partially confirms the previous findings. Considering the concealment of psychological abuse relative to physical abuse, more efforts to develop and implement the interventions to address psychological abuse effectively must be undertaken. In addition, the prevalence of sexual abuse in China is lower than that abroad [51–53], which may be due to cultural differences. On the one hand, because of the influenced of the traditional culture, Chinese women are unwilling to talk about sex-related topics, and even after being a victim of sexual abuse, they are less likely to seek help or talk to others because of the feelings of shame and the fearing of discrimination. On the other hand, although there was compulsive sexual behavior in couples, many women did not regard it as a type of domestic violence. This may lead to the underestimated prevalence of sexual abuse against women during pregnancy in China.

Because IPV during pregnancy has been increasingly recognized as an important risk factor for adverse health outcomes in women and their offspring, as well as its considerable prevalence, several studies have attempted to identify risk factors associated with being a victim of IPV during pregnancy [54–56]. In this study, subgroup analyses were performed to investigate risk factors that increased the risk of violence against women during pregnancy in a Chinese population. The results showed that only the variables “number of children” and “unplanned pregnancy” had significant effects on the prevalence of IPV during pregnancy (p < 0.05). Compared to women with more than two children, women with two or fewer children reported a significantly lower prevalence of IPV during pregnancy (6.8% vs 10.8%, p < 0.05). This finding is supported by the previous studies. In studies conducted by Deveci et al. [57] and Farid et al. [58], women who suffered violence from their partners during pregnancy had a significantly larger average number than those who had not (2.2 ± 1.9 vs 1.3 ± 1.4, 2.28 ± 1.41 vs 1.80 ± 1.18). In addition, a population-based, multicenter, cross-sectional household survey showed that compared to women who had only one child, women with two children and women with three or more children were at a 0.4- and 0.6-fold increased risk of IPV during pregnancy [59]. There findings suggest that the number of children may be a positive risk factor for women experiencing IPV during pregnancy. This may be explained by the possibility that when women had more children, the economic pressure perceived by their partners were greater, which could lead to a greater likelihood of contradiction or conflict in couples and may finally result in the occurrence of IPV. In addition, in the present meta-analysis, the prevalence of IPV during pregnancy in women with an unplanned pregnancy was significantly higher than women with a planned pregnancy (35.9% vs 11.6%, p < 0.05), suggesting that unplanned pregnancy may be a potential risk factor associated with experiencing violence during pregnancy. In the United States, a population-based study showed that the prevalence of IPV during pregnancy was significantly higher in women who experienced unplanned or untimely pregnancy than women with a clear planned pregnancy (15% vs 5%) [60]. Stewart et al. [61] found that compared to women with a planned pregnancy, women with an unplanned pregnancy had a 3-times higher risk of suffering IPV during pregnancy. In New Zealand, a survey of Pacific Islander families showed that women who experienced physical abuse were more likely to report an unplanned pregnancy than women who did not (68.7% vs 55.1%, OR = 1.78) [62]. It was suggested that in at least some abusive families, a woman’s need for contraceptives were unmet, and the atmosphere of fear and control caused by abusive relationships could limit the ability of women to control their fertility, which could finally lead to an unplanned pregnancy [50]. Disagreements between pregnant women and their partners regarding the acceptability of unplanned pregnancy, coupled with the impact of pregnancy on sexual life may lead to oral and emotional confliction and finally to the occurrence of violence during pregnancy.

Several studies demonstrated that there is a relationship between maternal and paternal alcohol use and the risk of IPV during pregnancy [55, 63–66]. Dunkle et al. [66] found that the prevalence of IPV during pregnancy in women with a drinking problem was 4.59-time higher than that in women who did not have a drinking problem during pregnancy, whereas a 10-fold increased risk was found in women who regularly consumed alcohol during pregnancy in a study by Olagbuji et al. [63]. Even after adjusting for confounding factors, women who drank during pregnancy were approximately 2-times as likely to be abused during pregnancy compared to women who did not drink [55]. Additionally, Ntaganira et al. [67] found a 3- and 2-fold increased risk of experiencing violence during pregnancy respectively, in women who had an occasional alcohol drinking partner and women who had a heavy alcohol drinking partner compared to women whose partner did not drink during pregnancy. Similar results were found by Fawole et al. [68] and Muhajarine et al. [69]. On one hand, drinking alcohol could affect cognitive and physical functions directly, which could lead to a decrease in a couple’s ability to solve conflicts in a peaceful way. On the other hand, excessive drinking could increase the financial burden on the entire family and cause troubles for raising children. Furthermore, it could increase the risk of infidelity, which would possibly lead to conflict between couples and the occurrence of IPV during pregnancy. However, in the present meta-analysis, there was no significant effect found between maternal alcohol use and the risk of IPV during pregnancy (p > 0.05) or between paternal alcohol use and the the risk of IPV during pregnancy (p > 0.05). However, there were only a few studies included about this issues and we could not exclude the possibility that the power of this test was too low to identify differences; therefore we do not suggest that maternal and paternal alcohol use has no influence on the prevalence of IPV during pregnancy in the Chinese population. This must be confirmed by a well-executed, national-wide study.

There were some limitations that must be considered. As with other systematic reviews and meta-analyses, significant heterogeneity was found in the prevalence estimates and was incompletely explained by the univariate meta-regressions analysis. In the present study, only the variable “sample source” explained part of the heterogeneity, which suggested that there might be other unknown factors that account for the variability between included studies. For example, none of the included studies reported the overall prevalence based on the national population. In the past, because of the bias and some degree of uncertainty that may be introduced by the selected population (e.g., sub-national or local samples) and settings (e.g., hospital-based vs population-based), studies that did not target the entire population were criticised. Previously, a review by Taillieu et al. [49] suggested that the characteristics of the population, such as age, could contributed to the range of prevalence estimates of IPV during pregnancy. However, we did not obtain adequate information for this. In addition, although extensive literature retrieval was preformed, the existence of non-indexed studies in the retrieved databases may led to the omission of some relevant studies. Moreover, although an attempt was made to minimize the possible bias in the process of literature searching with specific searches in major Chinese-English databases (including master and doctoral theses), there may be other unidentified studies. Fortunately, as denoted by the funnel plot and the Egger’s tests, publication bias was not anticipated because we obtained a certain proportion of data from unpublished studies (two theses [30, 32]). We also conducted a sensitivity analysis to assess the uncertainty assumptions on the pooled prevalence of IPV for the methodological quality of the included studies, which indicated the validity of the statistical calculations in the present meta-analysis.

## Conclusions

The prevalence of IPV during pregnancy based on evidence from existing observational studies is easily calculated and helpful for measuring the prevalence of IPV during pregnancy in a specific country. In particular, findings in the present study suggest that the prevalence of IPV during pregnancy is considerable in China and is one of the highest reported in Asia, which suggests that issues of violence against women during pregnancy should be included in efforts to improve the health of pregnant women and their offspring. In addition, a nationwide epidemiological study is needed to confirm the prevalence estimates and identify additional risk factors for IPV during pregnancy.

## Supporting information

S1 Checklist PRISMA Checklist. (DOC)

S1 File List of excluded references and reasons for exclusion. (DOC)
