# Supplementary material for: Prevalence of intimate partner violence (IPV) during pregnancy in China: A systematic review and meta-analysis
Source: PLoS One. 2017 Oct 2;12(10):e0175108. doi: 10.1371/journal.pone.0175108 (PMC5624577; doi:10.1371/journal.pone.0175108)
Supplement: S1 File — (DOC) [file pone.0175108.s002.doc]

**S1 File. List of excluded references and reasons for exclusion.**

| **References** | **Reason** |
| --- | --- |
| 1. Tian L, Liu X, Tian H. Adverse effects of demestic violence during pregnancy. J Ningxia Med Univ, 2015; 37(7): 854-57. doi: 10.16050/j.cnki.issn1674-6309.2015.07.042 | 2 |
| 2. Zhong JQ. The effect of demostic violence during pregnancy on the temperament and behavior of the preschool offspring. Thesis, Central South University, 2013. Available from: http://d.g.wanfangdata.com.cn/Thesis_y2423542.aspx | 5 |
| 3. Zhang Y, Zou SH, Zhang YL. Psychosocial factors related to domestic violence during pregnancy. Chinese Mental Health Journal, 2013; 27(02): 116-20. doi: 10.3969/j.issn.1000-6729.2013.02.007 | 1 |
| 4. Zhong JQ, Cao YP, Zhang YL. Impact of domestic violence during and after pregnancy on infants' development. Chin J Clin Psychol, 2013; 21(1): 91-94. | 2 |
| 5. Shi HL, Fan SQ, Li WT, Chang WJ, An LB. Influencing Factors and Psychosocial Reactions Status of Domestic Violence in Pregnant Women. Chin Gen Prac, 2013; 16(28): 3379-81. doi: 10.3969/j.issn.1007-9572.2013.28.027 | 2 |
| 6. Chan KL, Brownridge DA, Fong DYT, Tiwari A., Leung WC, et al. Violence against pregnant women can increase the risk of child abuse: A longitudinal study. Child Abuse Negl, 2012; 36(4): 275-84. doi: 10.1016/j.chiabu.2011.12.003 PMID: 22565038 | 1 |
| 7. Zhang Y, Zou SH, Cao YP, Zhang YL. Relationship between domestic violence and postnatal depression among pregnant Chinese women. Int J Gynaecol Obstet, 2012; 116(1): 26-30. doi: 10.1016/j.ijgo.2011.08.011 PMID: 22024214 | 1 |
| 8. Zhang Y, Zou SH, Cao YP, Zhang YL. Correlation of Domestic Violence and Postnatal Depression among Pregnant Women. Chin J Clin Psychol, 2012; 20(04): 506-09. | 1 |
| 9. Zhang Y., Zou SH, Cao YP, Zhang YL. Correlation of Domestic Violence and Postnatal Depression among Pregnant Women: The 2012 Conference on Mental Science in Shandong and Tianjin, Jinan, 2012[C]. Medical Association of Shandong Province & Tianjin Medical Association. | 2 |
| 10. Yen CF, Yang MS, Lai CY, Chen CC, Yeh YC, et al. Alcohol Consumption after the Recognition of Pregnancy and Correlated Factors among Indigenous Pregnant Women in Taiwan. Matern Child Health J, 2012; 16(2): 528-38. doi: 10.1007/s10995-011-0747-3 PMID: 21258959 | 3 |
| 11. Chan KL, Brownridge DA, Tiwari A., Fong DY, Leung WC, et al. Associating Pregnancy With Partner Violence Against Chinese Women. J Interpers Violence, 2011; 26(7): 1478-500. doi: 10.1177/0886260510369134 PMID: 20495098 | 4 |
| 12. Chan KO, Liu TT, Tiwari A., Leung WC, Fong D., et al. Intimate Partners' Violence against Chinese Pregnant Women:A Review of Studies in Mainland China and Hong Kong. Collection of Womens Studies, 2011; 2011(2): 87-94. doi: 10.3969/j.issn.1004-2563.2011.02.012 | 2 |
| 13. Zhang Y. Effect of domestic abuse during pregnancy on cognitive behavior of newborn: The Ninth National Academic Conference of psychiatry of the Chinese Medical Association, Guangzhou, 2011[C]. Chinese Medical Association. | 2 |
| 14. Chan KL, Tiwari A, Fong DY, Leung WC, Brownridge DA, et al. Correlates of in-law conflict and intimate partner violence against Chinese pregnant women in Hong Kong. J Interpers Violence, 2009; 24(1): 97-110. doi: 10.1177/0886260508315780 PMID: 18378806 | 1 |
| 15. Lau Y., Keung Wong DF, Chan KS. The impact and cumulative effects of intimate partner abuse during pregnancy on health-related quality of life among Hong Kong Chinese women. Midwifery, 2008; 24(1): 22-37. doi: 10.1016/j.midw.2006.06.010 PMID: 17196715 | 1 |
| 16. Zhang Y, Zhang YL, Zou SH, Zhang XH, Cao YP, et al. Correlation between domestic violence in pregnancy and the levels of plasma amino acids and cortisol in the neonates. Chin J Intern Med, 2008; 47(3): 209-12. doi: 10.3321/j.issn:0578-1426.2008.03.010 | 2 |
| 17. Mai DX, Wu BF, Lin LF. Correlative Study between Family Violence and Cerebral Palsy. Journal of Applied Clinical Pediatrics, 2008; 23(17): 1338-87. doi: 10.3969/j.issn.1003-515X.2008.17.033 | 2 |
| 18. Tiwari A, Chan KL, Fong D, Leung WC, Brownridge DA, Lam H, et al. The impact of psychological abuse by an intimate partner on the mental health of pregnant women. BJOG, 2008; 115(3): 377-84. doi:10.1111/j.1471-0528.2007.01593.x PMID:18190375. | 3 |
| 19. Zhang Y, Zhang YL, Zou SH. Psychosocial Risk Factors of Domestic Violence and Its Negative Influence on Pregnancy. Chinese Mental Health Journal, 2007; 21(12): 853-56. doi: 10.3321/j.issn:1000-6729.2007.12.014 | 2 |
| 20. Hu JZ, Liang WL, Wang XX, Xu SH. Investigation and analysis of domestic violence and its adverse effects among pregnant women within the jurisdiction: National Symposium on nursing management, nursing education and psychological nursing (Shenzhen) in 2007, Shenzhen, 2007[C]. Department of continuing education of the Chinese Medical Association & Chinese Nursing Association. | 2 |
| 21. Wu JL, Wang LH, Zhao GL, Zhang XS. Sexual abuse and reproductive health among unmarried young women seeking abortion in China. Int J Gynaecol Obstet, 2006; 92(2): 186-91. doi: 10.1016/j.ijgo.2005.10.021 PMID: 16356501 | 3 |
| 22. Lau Y. Does pregnancy provide immunity from intimate partner abuse among Hong Kong Chinese women? [Soc Sci Med](https://www.ncbi.nlm.nih.gov/pubmed/?term=Does+pregnancy+provide+immunity+from+intimate+partner+abuse+among+Hong+Kong+Chinese+women?), 2005; 61(2): 365-77. doi: 10.1016/j.socscimed.2004.12.002 PMID: 15893052 | 1 |
| 23. Ye ZH, Wang SY, Xiao XM, Fan L., Zhou YF, et al. Investigation on domestic violence in pregnant and postpartum women. Chin J Publ Heal, 2005; 21(8): 1012-13. doi: 10.3321/j.issn:1001-0580.2005.08.059 | 1 |
| 24. Ye ZH, Wang SY, Xiao XM, Ye C, Zhang Z, et al. A cross-sectional investigation on the domestic violence among pregnant women in Guangzhou and Shenzhen. Chinese Journal of Preventive Medicine, 2005; 39(3): 574-75. doi: 10.3760/j:issn:0253-9624.2005.03.026 | 1 |
| 25. Guo SF, Wu JL, Qu CY, Yan RY. Domestic abuse on women in China before, during, and after pregnancy. Chin Med J, 2004; 117(3): 331-36. PMID: 15043768 | 1 |
| 26. Guo SF, Wu JL, Qu CY, Yan RY. Domestic violence against women before, during and after pregnancy. Chin J Epidemiol, 2004; 25(01): 9-11. doi: 10.3760/j.issn:0254-6450.2004.01.004 | 1 |
| 27. Wu JL, Guo SF, Yan RY, Qu CY. Analysis on the related factors of domestic violence among women seeking abortion in northern cities. Chin J Publ Heal, 2004; 20(12): 1493-94. doi: 10.3321/j.issn:1001-0580.2004.12.047 | 1 |
| 28. Guo SF, Wu JL, Qu CY, Yan RY. Postpartum Abuse: Effect on Postpartum Depression. Chinese Mental Health Journal, 2003; 17(09): 629-31. doi: 10.3321/j.issn:1000-6729.2003.09.017 | 1 |
| 29. Liu GS, Shi GL, Wang LM, Lan FR. The family violence situation of 3025 children′s mothers and effect on the psychosomatic health of mothers and infants. Chin Gen Prac, 2003; 6(3): 226-28. doi: 10.3969/j.issn.1007-9572.2003.03.020 | 3 |
| 30. Leung TW, Leung WC, Chan PL, Ho PC. A comparison of the prevalence of domestic violence between patients seeking termination of pregnancy and other general gynecology patients. Int J Gynaecol Obstet, 2002; 77(PII S0020-7292(01)00596-31): 47-54. doi: 10.1016/S0020-7292(01)00596-3 PMID: 11929659 | 3 |

Reason for exclusion: 1. Duplicate publications; 2. Case-control studies, review papers and conference abstracts; 3. Having data that were not extractable; 4. With partners as research subjects; 5. With the fifth year after delivery as the assessment time.
